# Molecular Dynamics Study of Chitosan Adsorption at a Silica Surface

**DOI:** 10.1021/acs.jpcc.4c05821

**Published:** 2024-12-10

**Authors:** Magdalena Hudek, Karen Johnston, Karina Kubiak-Ossowska, Valerie A. Ferro, Paul A. Mulheran

**Affiliations:** † Department of Chemical and Process Engineering, University of Strathclyde, 75 Montrose Street, Glasgow G1 1XJ, U.K.; ‡ ARCHIE-WeSt, Department of Physics, University of Strathclyde, 107 Rottenrow East, Glasgow G4 0NG, U.K.; § Strathclyde Institute of Pharmacy and Biomedical Sciences, University of Strathclyde, 161 Cathedral Street, Glasgow G4 0RE, U.K.

## Abstract

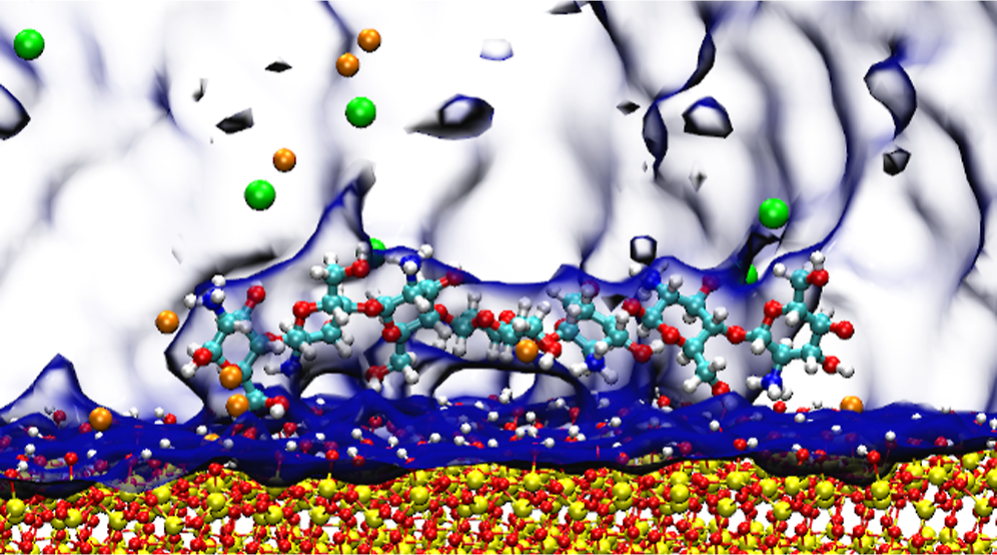

Chitosan is a nontoxic biopolymer with many potential biomedical and material applications due to its biodegradability, biocompatibility, and antimicrobial properties. Here, fully atomistic molecular dynamics simulations and enhanced sampling methods have been used to study the adsorption mechanism of chitosan oligomers on a silica surface from an aqueous solution. The free energy of adsorption of chitosan on a silica surface was calculated to be 0.6 kcal mol^–1^ per monomer in 0.15 mol L^–1^ aqueous solution, which is comparable to *k*_B_*T* at room temperature. The loading capacity of chitosan on the silica surface was found to be 0.094 mg m^–2^, and it is dominated by charge compensation. Furthermore, the hydrogen bonding between chitosan and silica was analyzed. The nitrogen and hydroxyl group oxygen chitosan atoms were found to be the main contributors to the hydrogen bonding between chitosan and silica. These findings have the potential to guide the experimental design of chitosan-coated silica nanoparticles for applications such as drug delivery or additives for biopolymer food packaging.

## Introduction

Chitosan is a polysaccharide with many desirable properties, most notably it is antimicrobial, nontoxic, and biodegradable,^1^ and thus has a wide range of uses in biomedical and food packaging applications.^2^ However, pure chitosan films have poor mechanical and moisture barrier properties^3^ and thus require further optimization to improve their utility. Chitosan–silica nanoparticle composites can be used as additives to enhance the mechanical and oxygen/water permeation barrier properties of films^4^ or to impart functionalization such as antimicrobial properties.^5^ Chitosan–silica composites have been studied in various forms, including silica–chitosan hydrogels for heavy-metal absorption^6^ and chitosan-coated silica nanoparticles for targeted chemotherapy drug delivery.^7^

**Figure 1:**
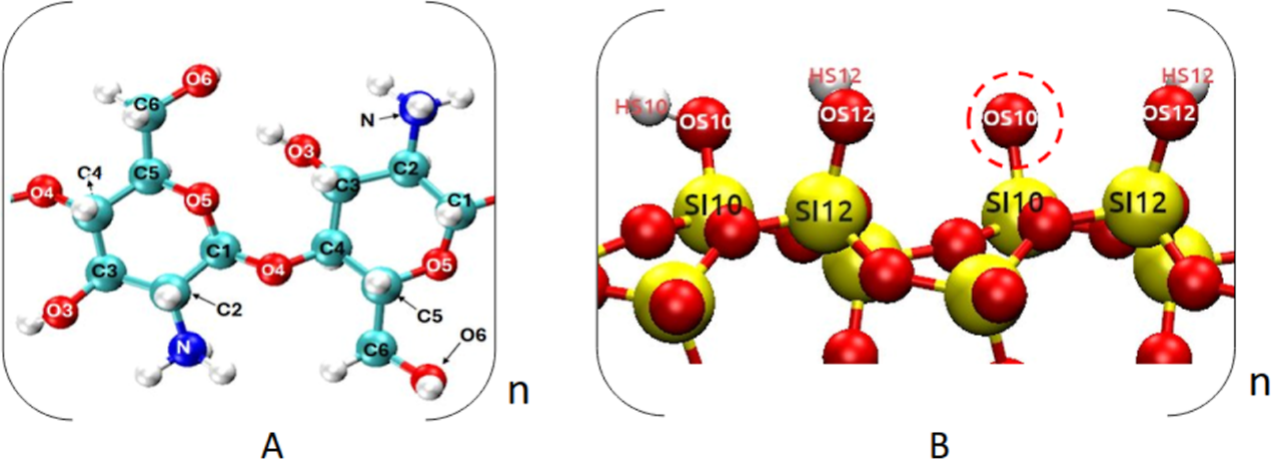
Schematic diagram for chitosan (A) and the silica surface (B) where atoms are labeled for reference. The dashed red circle in (B) highlights deprotonated surface oxygen. The colors are assigned according to the atomic species: cyan—carbon, red—oxygen, blue—nitrogen, yellow—silicon, and white—hydrogen.

Chitosan is produced by the deacetylation of chitin, which is the second most abundant naturally occurring polysaccharide, and consists of *N*-β(1–4) linked glucosamine monomers as shown in Figure 1A. Structurally, chitosan is very similar to cellulose, with the only difference being the amino group (−NH_2_) at the C2 carbon atom, whereas cellulose has a hydroxyl (−OH) group at this position. The presence of an amino group makes chitosan polycationic, unlike other naturally occurring biopolymers, which are neutral or polyanionic. Consequently, chitosan is soluble in weak aqueous acids (e.g., acetic, lactic, and oxalic) below a pH of 5.4.

The silica–water interface is characterized by the presence of the electric double layer (EDL). The EDL consists of ions that screen silica’s negatively charged surface. The extent of the EDL can be approximated by the Debye length (λ_D_).^8^ It depends on the concentration (*c*_*j*_) and the valency (*z*_*j*_) of the ions in the bulk, the temperature (*T*), and is given by
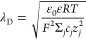
1where *F* is the Faraday constant, *R* the ideal gas constant, ε_0_ is the permittivity of the free space, and ε is the dielectric constant. The *j* subscript denotes the ionic species present in solution.

Understanding the interface between the silica surface and chitosan, and the mechanism of chitosan adsorption, is crucial for ensuring particle stability and fine-tuning the properties such as particle charge, size, and bioactivity. Several studies have used experimental methods, for example, quartz crystal microbalance and UV–vis spectra, to explore the mechanism of chitosan adsorption from solution onto silica surfaces.^9,10^ A Fourier transform infrared spectroscopy study^11^ showed the presence of hydrogen bonding. While these studies provide useful information such as chitosan adsorption density and chitosan layer thickness, these experimental methods cannot provide an atomistic level of detail of the adsorption process. Here, we complement these previous studies by using in silico methods based on molecular dynamics (MD) simulations. We aim to provide insight and understanding of the dynamics of chitosan adsorption at the molecular level, in order to further improve and optimize chitosan-coated silica nanoparticles tailored to specific applications.

While MD simulations provide atomic and molecular resolution, sampling of the entire space-phase landscape can be difficult. Enhanced sampling methods such as steered MD (SMD) and umbrella sampling (US) can be used to calculate free energies of adsorption and the dynamics of rare events. Combinations of standard MD and enhanced sampling methods provide powerful tools for understanding fundamental material and interaction properties and thus can be used to guide the design of new materials.^12^

SMD simulations are widely used to study various biomolecular systems, including protein stretching, protein–ligand binding, and drug binding affinity,^13^ as well as polymer and protein binding to inorganic surfaces.^14,15^ The advantage of SMD is that it can be compared to atomic force microscopy and optical tweezer experiments while providing an atomistic level of detail that cannot be achieved experimentally. US has previously been successfully used to calculate the free energy profile of different molecules on the silica surface, such as DNA,^16^ catechols,^17^ and peptides.^18^

**Figure 2:**
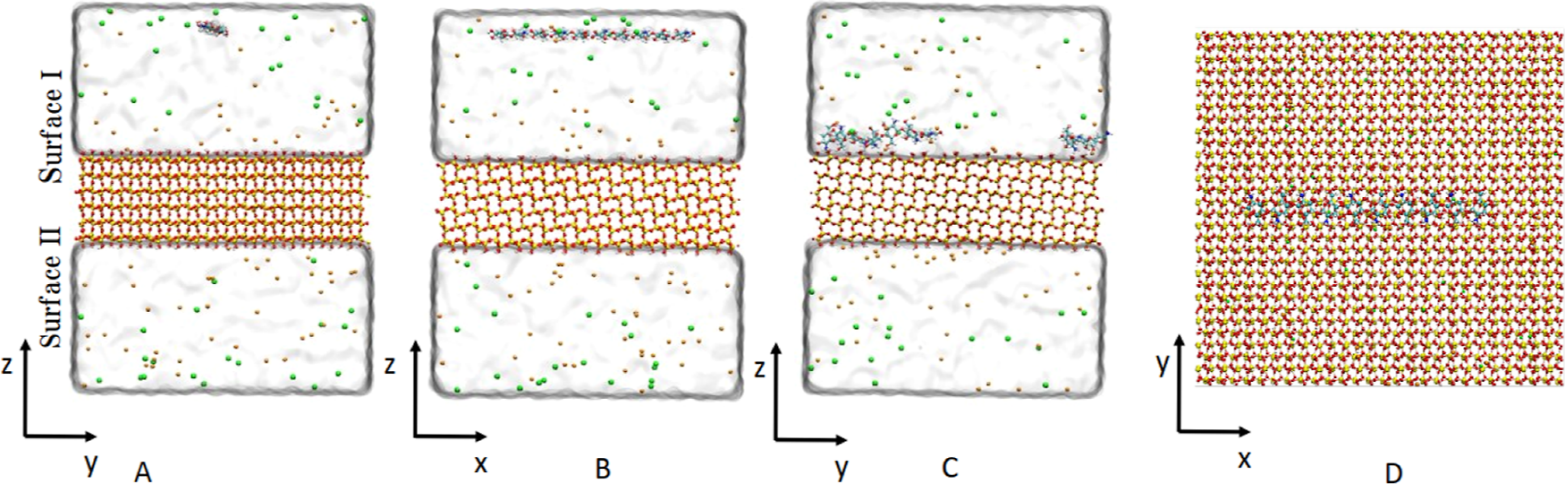
A typical system is shown at the beginning (A,B,D) and at the end (C) of the simulation. The system is periodic in all three dimensions. The colors are assigned according to the atomic species: cyan—carbon, red—oxygen, blue—nitrogen, yellow—silicon, white—hydrogen, green—chloride ions, and orange—sodium ions. Water is omitted from D for clarity.

In previous work, we used SMD and US to study the interaction between chitosan and chitin crystals in aqueous solution and found that the adsorption process is driven by van der Waals forces and coordinated via hydrogen bonding.^19^ In this work, we build on this modeling framework to study the interactions of chitosan in aqueous solution with silica surfaces. SMD was used to explore the adsorption of chitosan oligomers from aqueous solution to a model silica nanoparticle (Si-NP) (2 0 −2) surface, as illustrated in Figure 2. Here, the spherical Si-NP has been modeled as a flat surface. This approximation is valid for nanoparticles with a size of ≈100 nm, where the curvature of a surface segment with a size of approx 7.4 nm by 7.4 nm can be neglected. This approximation, however, would not be valid for very small NPs (size on the order of ≈10 nm). The oligomer-by-oligomer adsorption was studied as well as simultaneous multioligomer adsorption. US was used to calculate the free energy of adsorption of chitosan onto the Si-NP surface and determine the mechanism of adsorption. These simulations can be used both to aid interpretation of experiments and, in the future, for design optimization for bespoke applications of the functionalized nanoparticles.

## Methods

### System Setup and General Simulation Protocol

A silica slab with a (2 0 −2) surface and size (*x* = 7.49 nm, *y* = 7.44 nm, and *z* = 2.6 nm) with periodicity in the *xy* plane was constructed using the CHARMM-GUI^20^ nanomaterial modeler^21^ with an α-cristobalite structure and a surface silanol (Si–OH) group concentration of 4.7 nm^–2^. The slab exposes two chemically identical surfaces to the solution (surfaces I and II) that are able to adsorb oligomers. Chitosan oligomers were constructed using an in-house Python code.^19^ The silanol deprotonation level was 6.66% (0.31 nm^–2^) and the chitosan was fully protonated to mimic pH ≈ 5. INTERFACE^22^ and CHARMM36^23^ widely used force fields were used to model the silica and chitosan, respectively. We have successfully used the CHARMM36 force field in our previous work.^19^ The INTERFACE force field accurately reproduces the silica–water interface.^24^ The structures were combined using VMD^25^ to obtain the desired initial system configuration. The system was then solvated with TIP3P^26^ water, and Na^+^ (or Ca^2+^) and Cl^–^ ions were added to neutralize the system and set the bulk solution concentration to 0.15 mol L^–1^. The parameters for the water model and ions were obtained from the CHARMM36 force field and have been shown to reproduce experimental phenomena correctly.^27^

All the simulations were performed using Gromacs^18^ 2022.2 software, with the inputs prepared using the ParmEd code.^28^ The analysis was performed using Gromacs built-in tools, VMD, and MDAnalysis,^29^ while the plots were made using Gnuplot.^30^

The general simulation protocol was as follows. The system was initially minimized for 5000 steps using the steepest descent method, with nonsolvent molecules restrained using harmonic potentials. The water molecules and ions were then equilibrated for 0.5 ns using the Berendsen thermostat at 300 K with 0.1 ps time constant for coupling and the Parinello–Rahman barostat with anisotropic pressure coupling at 1.0 bar with 0.2 ps time constant for coupling and compressibility of 2.5 × 10^–6^ bar^–1^ in the *x* and *y* directions and 4.5 × 10^–5^ bar^–1^ in the *z* direction. After initial equilibration, the system was simulated using the Nose–Hoover thermostat at 300 K with 1.0 ps time constant for coupling, the Parinello–Rahman barostat at 1 bar with anisotropic pressure coupling with 0.2 ps time constant for coupling and compressibility of 2.5 × 10^–6^ bar^–1^ in the *x* and *y* directions and 4.5 × 10^–5^ bar^–1^ in the *z* direction, and 2 fs time step integration. The electrostatics were calculated using particle mesh Ewald with a 1.2 nm cutoff. The LINCS constraint algorithm was used to restrain hydrogen bonds. Periodic boundary conditions were used, so that the silica slab is infinite in the *x* and *y* directions, with the solution sandwiched between silica slabs. To ensure the reproducibility of our simulations, all the inputs are freely available in the associated data set (see Data Availability Statement).

### Chitosan Adsorption

A number of systems were created for standard MD simulations. These are described below (with the quantitative component summary provided in Table S1) and a representative system is shown in Figure 2. Here, a single chitosan 6-mer (0.9 kDa) or 10-mer (1.6 kDa) was placed 3 nm above the silica surface, and the system was further simulated as described in the aforementioned general protocol for 200 ns. The entire protocol was repeated twice with newly generated silica slabs to ensure random silanol deprotonation sites. All the production trajectories, carried out in triplicate, were analyzed and they gave statistically similar results, therefore one representative trajectory obtained for each system was used as an exemplar and is described in detail in the Results and Discussion section.

To further study the loading capacity, which is the amount of chitosan capable of adsorbing to the silica surface, simulations were conducted with multiple chitosan oligomers present in the system. This was carried out using two separate methodologies: (i) inserting oligomers into the system one by one and (ii) adding all oligomers simultaneously at the beginning.

In the first methodology, oligomers were added one by one to the system. The system was initially set up in the same way as that for the single-oligomer adsorption simulations. However, in this case, the simulation was stopped after 100 ns. Following this, the water and ions were removed, and an additional single oligomer was placed 3 nm above the surface, bringing the total number of chitosan chains in the system to two. The system was then resolvated, and sodium chloride was added as described in the main simulation protocol. The production run was again 100 ns. The same process as before was repeated two more times, bringing the total number of chitosan oligomers in the system to four. This system was then simulated for 800 ns.

In the second methodology, eight 10-mer chitosan oligomers were placed 3 nm above the silica surface in a 4 × 2 arrangement with 1.5 nm spacing between the oligomers. The system was then prepared as described in the general protocol. An additional system was created which in which Ca^2+^ and Cl^–^ ions were added to neutralize the system and bring the bulk solution concentration to 0.15 mol L^–1^ to study the effects of the cation valency on the adsorption process. Lastly, a system of comparable size was created which contained only a solvated silica slab, neutralized with NaCl to 0.15 mol L^–1^ concentration. This system served as a control for the density profile calculations. All three systems were equilibrated using the main simulation protocol as above with a production run of 700 ns for each. In total, 9 different systems were simulated using standard MD totalling 3.4 μs.

### Adsorption Free Energy

SMD and US were performed using both a single 6-mer and a single 10-mer chitosan oligomer with a constant 1 nm ns^–1^ pulling velocity and harmonic constant *k* = 100 kcal mol^–1^ nm^–2^. SMD was performed on the systems with a single chitosan oligomer already adsorbed to the surface (after 200 ns of standard MD simulation time). The C4 atom (Figure 1) in the first monomer of the chitosan chain was pulled and later used to define the distance collective variable in the US simulations.

The SMD trajectory was analyzed and used to obtain snapshots for the US windows. In total, 53 and 72 windows were utilized with window spacing of 0.1 nm for the 6-mer and 10-mer systems, respectively (see umbrella histograms in Figures S4 and S5 in Supporting Information). A harmonic constant of *k* = 250 kcal mol^–1^ nm^–2^ was used. Each window was simulated for at least 40 ns, while the simulation time for any windows with uneven sampling was extended up to 80 ns. The total simulation times per US set were 1.34 and 3.66 μs for the 6-mer and 10-mer systems, respectively. The collective variable was defined as the distance between the C4 atom used in the SMD and the silica surface, with the surface defined as the arithmetic mean of the *z* positions of the silanol oxygen atoms. The free energy curves were calculated using the Gromacs built-in function which employs the weighted histogram analysis method.^31^ The US results were carefully analyzed to assess the reliability of the calculations.

### Analysis

Hydrogen bond analysis was performed using VMD’s “measure hbonds” command with cutoffs of 0.35 nm and 30° as predefined in VMD, following our previous study.^19^ Partial densities were calculated using the gmx density command, using 140 slices. Unless stated otherwise, in the time-averaged calculations the initial 20 ns of the simulation were omitted to ensure only portions of the simulation after the initial adsorption process was sampled.

## Results and Discussion

### Single Chain Adsorption Dynamics

**Figure 3:**
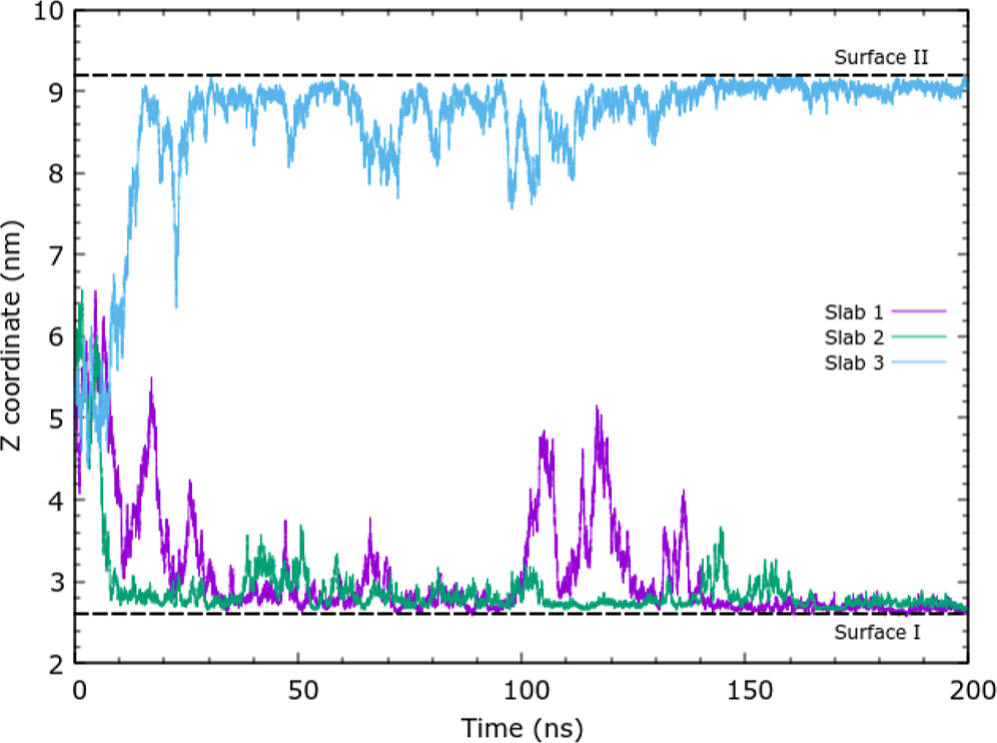
*z* components of chitosan 10 mer COM without periodic boundary condition wrapping. Three independent simulations (triplicates) are reported (labeled slab 1, 2, or 3).

The simulations of a single chitosan oligomer were performed in triplicate. The trajectories exhibit similar behavior, so we describe one 10 mer trajectory in detail as a representative. The chitosan oligomer readily approached the silica surface within 1 ns, initially with an orthogonal orientation to the surface. Through the next 10 ns of the simulation, the oligomer adopted an orientation parallel to the silica surface. Once adsorbed, the oligomer stayed on the surface for tens of nanoseconds before partially desorbing and moving parallel to the surface. Thus, the oligomer remained relatively mobile on the silica surface but did not desorb back into the bulk solution. The *z* components of the center of mass (COM) of the 10 mer oligomers for all three replicates are shown in Figure 3.

As described in the Methods, the initial structure of the triplicates differs in the random distribution of the deprotonated silanol groups on the silica slab surface. One limitation of the MD simulations is the inability to correctly model the protonation–deprotonation process of the silanol groups. Nevertheless, this is an equilibrium process, so on average the system will have the same number of protonated and deprotonated groups but will have different distributions. Simulations with different random deprotonation patterns enable us to see if the distribution of these sites makes a statistical difference to the adsorption process. In the COM plots shown in Figure 3, due to the use of unwrapped coordinates, surface I is at ≈3 nm and surface II at ≈9 nm in the figure. Chitosan is able to adsorb to either surface I or surface II as they are chemically the same. The 6-mer chitosan oligomers exhibit similar adsorption dynamics with the *z* component of the COM shown in the Supporting Information. Complete desorption into the solution is occasionally seen in the 6 mer adsorption simulation, with the chain subsequently readsorbing to the surface.

**Figure 4:**
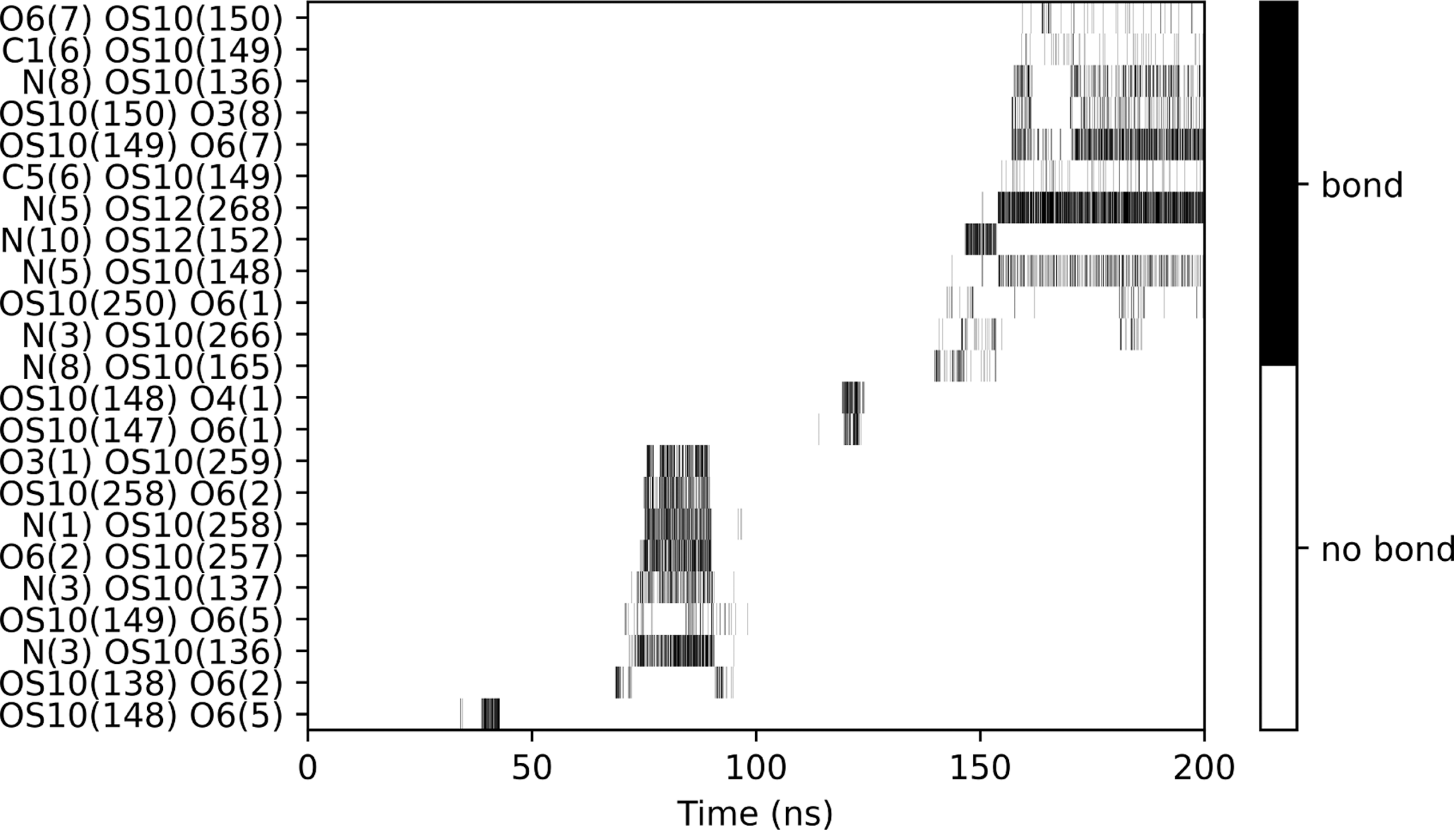
Chitosan–silica hydrogen bond formation and breaking during the adsorption process. The pairs are listed in donor–acceptor order. The atom names are as illustrated in Figure 1 with the number in the bracket referring to the residue number.

It is widely assumed that the adsorption process is driven by electrostatic interactions. The silica surface is negatively charged at a pH of 5, with deprotonated silanol groups (Si–O^–^). At the same pH, chitosan’s amino groups are protonated (−NH_3_^+^) making chitosan act as a weak polycationic electrolyte. However, hydrogen bonds are also important for adsorption. While it is not possible to distinguish individual energy contributions, hydrogen bonds arising from the electrostatic and Lennard-Jones terms can be monitored. Figure 4 shows the hydrogen bonds between the chitosan 10 mer and the silica slab during the simulation. The hydrogen bonds tend to break and form during the adsorption process. The highest number of simultaneous hydrogen bonds occurs after 150 ns of simulation, where the COM *z*-coordinate plateaus (Figure 3). The chitosan atoms that contribute the most to hydrogen bonding are O6 and N atoms, which are on opposite sides of the carbohydrate ring (see Figure 1). Moreover, due to chitosan’s helical conformation, which is typical of 1–4 linked polysaccharides in aqueous solution, two neighboring O6 or N atoms never contribute to hydrogen binding at the same time.

### Chitosan Desorption from the Surface Using SMD

**Figure 5:**
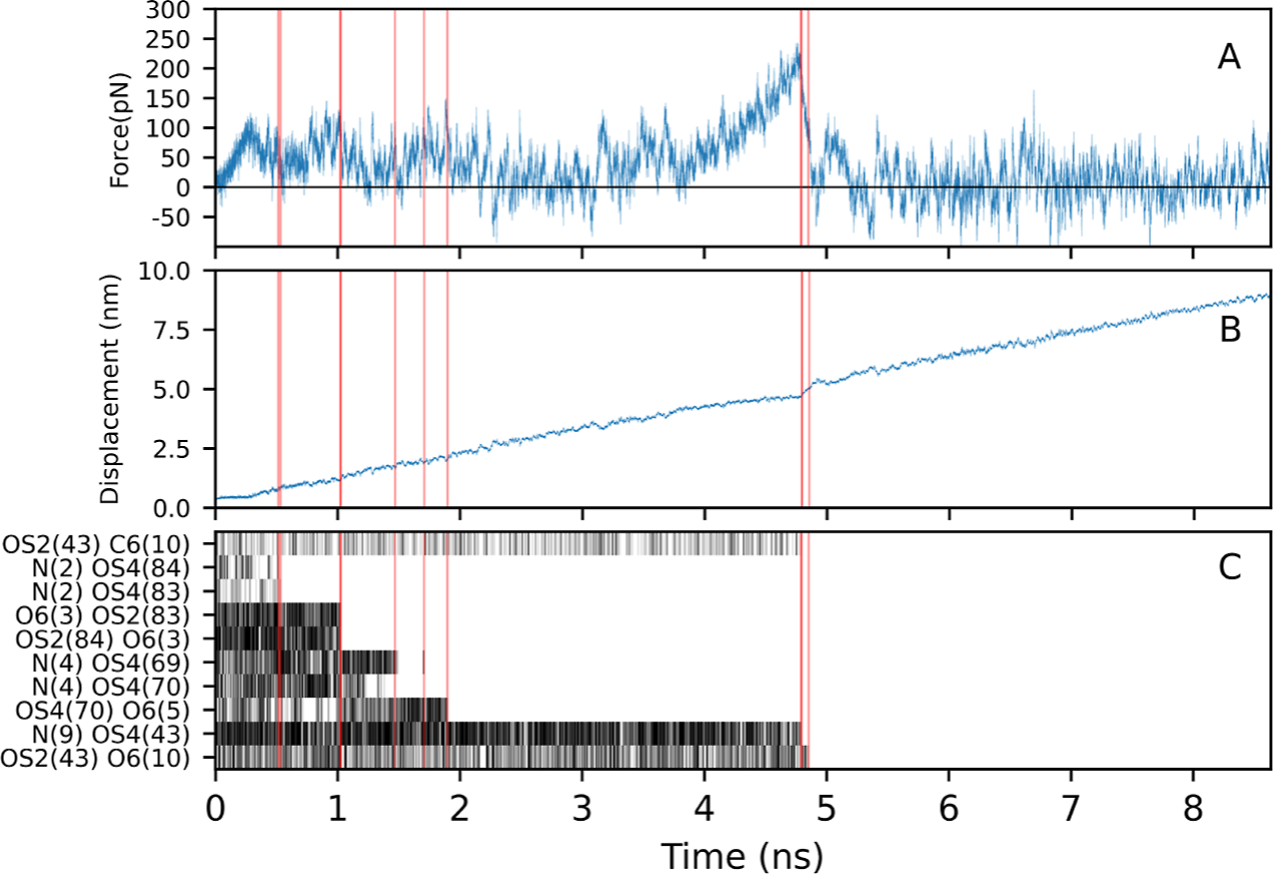
SMD analysis for a 10 mer chitosan oligomer pulled from the silica surface showing (A) force and (B) displacement curves. (C) Shows when key hydrogen bonds exist, with red vertical lines indicating the time of bond breaking. The pairs are listed in donor–acceptor order. The atom names are as illustrated in Figure 1 with the number in the bracket referring to the residue number.

SMD was used to qualitatively analyze hydrogen bond making and breaking between the chitosan and silica surfaces, as well as to obtain the initial structures for US windows. Figure 5 shows the analysis for the 10-mer chitosan SMD simulation, where the adsorbed oligomer is pulled directly up from the silica surface in the normal direction. The force curve shown in Figure 5A shows large variations in the force during the initial stage (0–2 ns) of the pulling. Once the chain is almost entirely pulled from the surface at time ≈4.9 ns, the force begins to show less large variations, with the mean value remaining greater than zero. After the chain exits the EDL, the force curve consists solely of noise with approximately zero mean, so that no further net work is done by the pulling force. The corresponding displacement curve of the pulled C4 atom is shown in Figure 5B.

Figure 5C shows the breaking of the key hydrogen bonds between the chitosan chain and the silica surface. These events are indicated by vertical red lines and correspond to the force drops observed in Figure 5A. The main chitosan atoms responsible for chitosan-to-silica hydrogen bonds are the nitrogen of the amino group and the O6 atom (see Figure 1). The nitrogens from monomers 2, 4, and 9 and the O6 atoms from monomers 3, 5, and 10 are the main contributors. The silanol oxygens (OS2, OS4) can act as both donors or acceptors in hydrogen bonding due to being part of the silanol functional group (Si–OH), with OS4(43) oxygen being the only deprotonated oxygen relevant for hydrogen bonding. Thus, this atom can be only an acceptor. This hydrogen bonding pattern is consistent with our previous observations in the spontaneous adsorption section. The harmonic constant (*k*) value used in here is too small for the stiff spring approximation to apply and thus we do not calculate free energy curves directly from our SMD trajectories,^32^ using instead US.

### Free Energy of Adsorption

**Figure 6:**
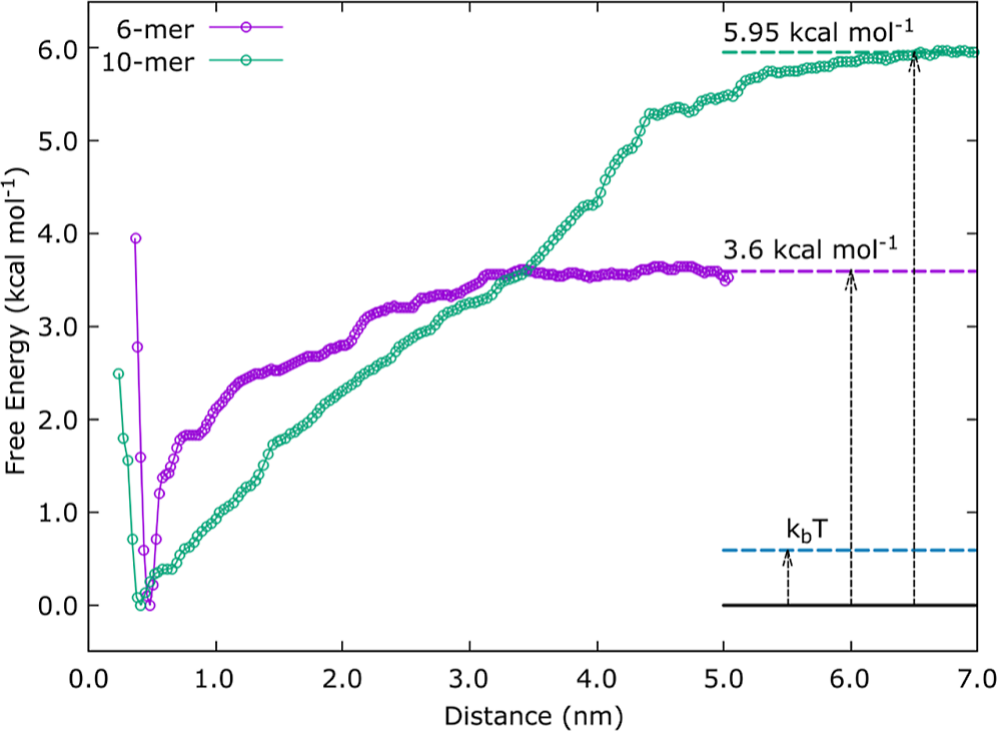
Free energy curves for the 6-mer and 10-mer adsorption.

The free energy profiles for the 6-mer and 10-mer chitosan oligomer adsorption processes are shown in Figure 6 and were calculated using US. The distance indicated in the energy curve graph is the reaction coordinate used in the US and is equal to the distance between the C4 atom of the first monomer and the silica surface. The other parts of the chain are not restrained by the harmonic potential and are thus allowed to move freely.

The energy minimum occurs at a 0.4 nm distance, which corresponds to the adsorbed state. After the minimum, both curves are relatively smooth, indicating no significant intermediate energy barriers to the adsorption process. Here, it is important to consider the length of each chain in evaluating the free energy curves. The end-to-end distances are 5.20 and 3.0 nm for completely straight 10-mer and 6-mer oligomers, respectively. Hence, the chitosan oligomer is fully desorbed only at a distance greater than the end-to-end chain length added to the thickness of the EDL, which can be approximated by the 0.8 nm Debye length for 0.15 mol L^–1^ NaCl concentration. Thus, by simple addition we expect the free energy curves to reach a plateau of around 6.0 and 3.8 nm, respectively, for the 10-mer and 6-mer, consistent with Figure 6.

The free energy of adsorption of chitosan oligomers to the silica surface can be calculated as the difference between the adsorbed state and the solvated state, which is characterized by the free energy curve reaching a constant value. The free energy of adsorption has thus been calculated to be 3.60 ± 0.46 and 5.95 ± 0.25 kcal mol^–1^ for the 6-mer and 10-mer, respectively. For both oligomers, this equates to approximately 0.6 kcal mol^–1^ per monomer. Thus, it might be concluded that the chitosan with higher molecular weight would exhibit stronger adsorption to the surface as has been experimentally shown by Matusiak et al., who measured the adsorption of chitosan with three different molecular weights on Si NPs using the ninhydrin method.^10^ Furthermore, as illustrated in Figure 6, *k*_B_*T* ≈ 0.6 kcal mol^–1^ at *T* = 300 K, which explains why individual monomers repeatedly adsorb and desorb from the silica surface during the standard MD simulations, giving the adsorbed oligomer mobility without complete desorption.

**Figure 7:**
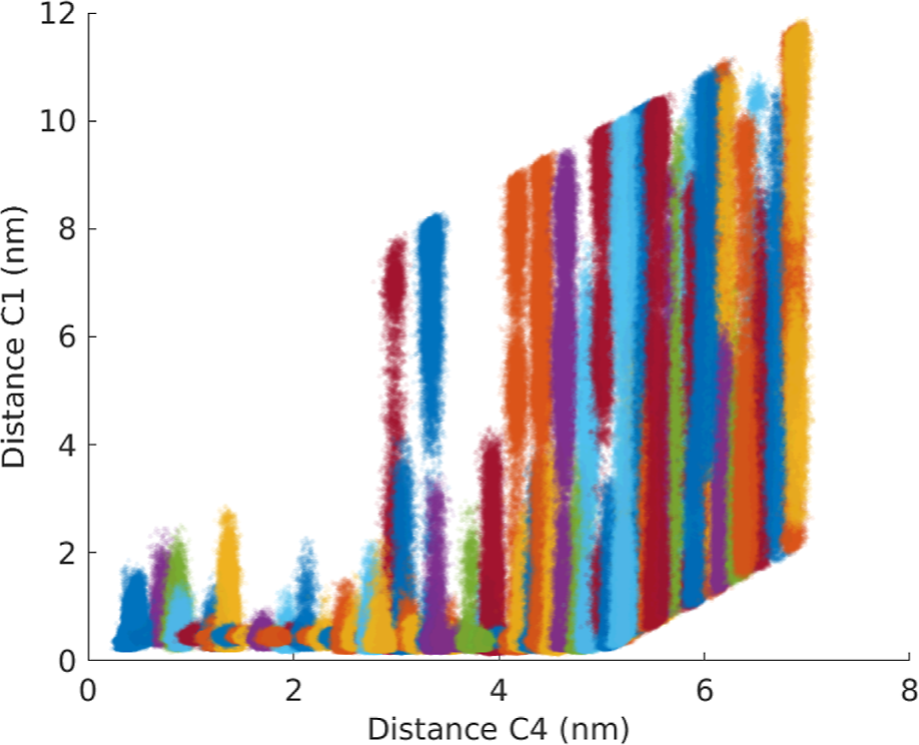
Constrained reaction coordinate (distance C4) and unconstrained orthogonal (distance C1) coordinate plot for 10 mer US set.

The US method cannot be directly evaluated for convergence; therefore, extensive indirect methods are employed instead. First, the histogram overlaps are evaluated, which are presented in Supporting Information (Figures S4 and S5) and show good overlap for all the windows. Another, often overlooked, property of the US histograms is the shape of each “umbrella” which should have a fairly symmetrical shape, indicating adequate sampling in the window. In cases where very asymmetrical umbrellas were observed, the simulation was extended until a more symmetrical shape was achieved to ensure adequate sampling. Additionally, the distance between the end C1 atom (at the opposite side of the oligomer to the C4 atom) and the silica surface was tracked to observe sampling of the phase space by the so-called orthogonal reaction coordinate, which is also responsible for US convergence. Figure 7 shows that the two distances display good overlap along both the *x* and *y* directions, which is indicative of good convergence of the calculated free energy profile.

Figure 7 also enables the visualization of the adsorption dynamics of the chitosan oligomer. As the C4 (constrained) distance is increased, the C1 (unconstrained) distance remains small until the C4 distance reaches 3 nm, indicating that chitosan remains partially adsorbed for those windows. For C4 distances between 3 and 4 nm, chitosan can either remain partially adsorbed or fully desorb, which depends on whether the initial configuration for the particular window has an energetically favorable position toward adsorption. Multiple windows for this range of C4 distances ensure good overlap and thus sufficient sampling of the conformational space. For C4 distances beyond 4 nm, chitosan fully desorbs and explores the full conformational space.

### Multiple Chain Adsorption Dynamics

To determine the maximum adsorption capacity of chitosan to the silica surface, we studied systems with multiple 10 mer chitosan oligomers present. The chitosan oligomers were initially placed into the solution 3 nm above the surface of the silica slab. Due to the periodicity of the system in the *z* direction, the chitosan was able to access both surface I and surface II, which have the same structure and deprotonation level. The oligomers were placed closer to surface I, so that the maximum level of oligomer adsorption is reached for that surface. However, upon reaching equilibrium, the same level of adsorption is observed for both surfaces.

In the systems where the chitosan oligomers were added one by one to the system, totalling four oligomers in the system, the number of adsorbed oligomers per surface was two for surface I and one for surface II. The oligomer that was added last to the simulation adsorbed to surface II for about 65 ns, but then it desorbed again (see Supporting Information Figure S2 for the COM plots). It remained in solution at 800 ns when the simulation was terminated.

In the systems where eight 10 mer chitosan oligomers were added simultaneously, the behavior of the oligomers depended on the salt used. As before, in the system solvated with sodium chloride, two chitosan oligomers adsorbed per surface and the other four oligomers remained in solution, which is equivalent to an adsorbed concentration of 0.35 monomers nm^–2^ (0.094 mg m^–2^). This is in agreement with the experimental study by Tiraferri et al.^9^ that used a quartz crystal microbalance with dissipation monitoring (QCM-D) and found adsorption of 0.083 mg m^–2^ at a pH of 4 and 100 mM NaCl concentration. They also measured the thickness of the adsorbed chitosan layer to be 0.6 nm at a pH of 4, which corresponds to the width of a single chitosan monomer in the hydrated conformation adsorbed to the surface in our simulations.

**Figure 8:**
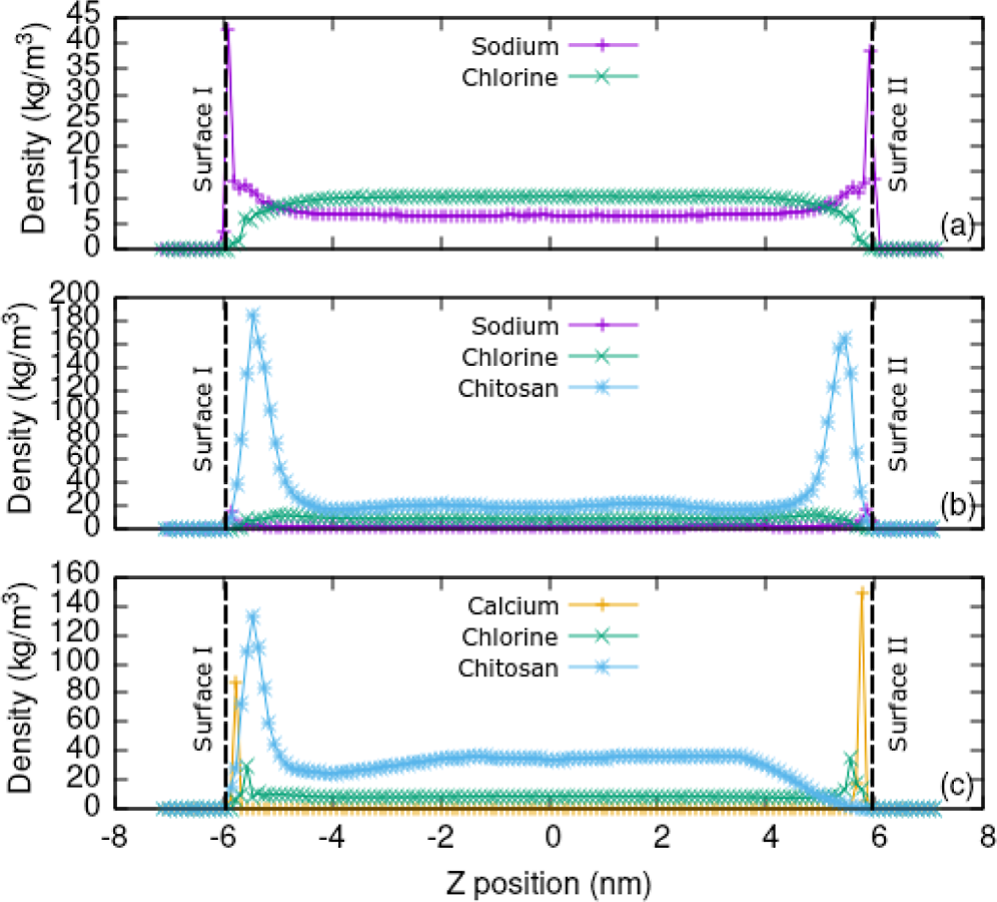
Partial density profiles in various simulations. (a) Silica slab only plus NaCl solution, (b) with eight 10 mer chitosan oligomers, and (c) with chitosan but with sodium replaced by calcium ions.

Figure 8 shows the time-averaged density profiles of different species in various simulations relevant to the eight 10 mer system. Here, the density was calculated in slices taken parallel to the silica surface (across the *x*–*y* plane) at different values of *z*, with the *z* axis being normal to the silica surfaces. The negatively charged silica surface is screened by the ions present in the solution, which form the EDL, and this can be seen in the Na^+^ cation density profile shown in Figure 8a when no chitosan is present. As weakly charged electrolytes, the chitosan oligomers compete with cations for silica adsorption sites. Each chitosan monomer contains a positively charged amino group (–NH_3_^+^), which has a +1*e* charge. This is the same valency as the sodium ions and hence chitosan is able to displace sodium from the silica surface, as can be seen by the lower sodium peak heights in Figure 8b when the chitosan oligomers are adsorbed.

In the system where the sodium ions were replaced by calcium ions, which have a +2*e* charge (Figure 8c), chitosan was not able to displace the calcium ions. Thus, chitosan is only able to adsorb to the deprotonated silanol sites which are unoccupied by calcium. There are 30 calcium ions and 36 deprotonated silanol groups which leave 6 deprotonated sites free for chitosan adsorption. In the density profile, it can be seen that the concentration of Ca^2+^ ions in bulk is zero and that some chitosan adsorption occurs at surface I, due to it having uncompensated deprotonated silanol and chitosan initial position closer to surface I. This shows that charge compensation is a dominant feature of the chitosan adsorption process that ultimately dictates the density of the adsorbed chitosan layer and furthermore that this can be controlled by choice of solute ions.

## Conclusions

We have used MD simulations and enhanced sampling methods to simulate chitosan oligomer adsorption on a model silica surface. The dynamics were studied of adsorption of a single chitosan oligomer from aqueous solution with 0.15 mol L^–1^ NaCl concentration, which corresponds to physiological conditions (or a technological application with a high salt concentration) at a pH of 5. The chitosan readily adsorbed to the silica surface, although it remained mobile on the surface.

Hydrogen bond formation and breaking was studied throughout the simulation, and hydrogen bond breaking was further analyzed using SMD. The chitosan atoms which contributed to hydrogen bonding were the nitrogen atom from the amino group and the O6 atom. To further quantify the strength of adsorption, US was used to calculate the chitosan oligomer free energy of adsorption to the surface in aqueous solution with a NaCl concentration of 0.15 mol L^–1^. The calculated energies were 3.60 ± 0.46 and 5.95 ± 0.25 kcal mol^–1^ for 10-mer and 6-mer oligomers, respectively, which is ≈0.6 kcal mol^–1^ per monomer. This is comparable to *k*_B_*T*, which explains why chitosan oligomers remain mobile at the silica surface.

The simulations showed that on average 0.35 monomers adsorb per nm^2^ of the silica surface (a chitosan mass density of 0.094 mg m^–2^) with NaCl salt, which is in good agreement with experimental studies, and aligns with the deprotonated silanol density (0.31 per nm^2^). In addition, we found that the chitosan layer thickness reported in the literature corresponds to the thickness of a single hydrated chain. We also demonstrated the importance of salt used in the simulations and found that divalent salts out-compete chitosan for potential absorption sites and thus prevent chitosan from adsorbing to the silica surface.

Our results provide insight into fundamental interactions between chitosan and silica surfaces. This can be used as a guide for future experimental design, with the concept of charge compensation and ion valency being used to control adsorbed densities. The optimization of chitosan-coated silica nanoparticles could lead to more effective targeted drug delivery with lower cytotoxicity or could be used to optimize functionalized antimicrobial food packaging.

## Data Availability

All data underpinning this publication are openly available from the University of Strathclyde KnowledgeBase at 10.15129/95d72512-3f2b-4b64-8163-d60c5842de89. In addition, the code used to generate chitosan oligomers is available at https://github.com/mhudek/generate_cht.

## Supporting Information Available

Tables summarizing system components and additional figures describing the adsorption process and US histograms (PDF)
