# Supplementary material for: Molecular Dynamics Study of Chitosan Adsorption at a Silica Surface
Source: J Phys Chem C Nanomater Interfaces. 2024 Dec 10;128(50):21531–8. doi: 10.1021/acs.jpcc.4c05821 (PMC11664576; doi:10.1021/acs.jpcc.4c05821)
Supplement: Supplementary file 1 — jp4c05821_si_001.pdf [file jp4c05821_si_001.pdf]

Supporting Information: Molecular Dynamics Study of Chitosan Adsorption at a Silica Surface

Magdalena Hudek,[†] Karen Johnston,[†] Karina Kubiak-Ossowska,[‡] Valerie A. Ferro,[¶] and Paul A. Mulheran[*,†]

†*Department of Chemical and Process Engineering, University of Strathclyde, 75 Montrose St, Glasgow G1 1XJ, UK*

‡*ARCHIE-WeSt, Department of Physics, University of Strathclyde, 107 Rottenrow East, Glasgow G4 0NG, UK*

¶*Strathclyde Institute of Pharmacy and Biomedical Sciences, University of Strathclyde, 161 Cathedral St, Glasgow G4 0RE, UK*

E-mail: paul.mulheran@strath.ac.uk

Standard MD simulations

Table S1: Summary of standard MD simulation systems

Chitosan	Silica slab no.	N(water)	N(Na^+)	N(Cl^-)	N(Ca^{2+})
6-mer	1	12813	66	36	0
6-mer	2	13069	67	37	0
6-mer	3	13084	67	37	0
10-mer	1	14833	68	42	0
10-mer	2	13759	65	39	0
10-mer	3	13852	65	39	0
8 × 10-mer	1	21529	61	105	0
8 × 10-mer	1	21555	0	104	30
-	1	22208	141	105	0

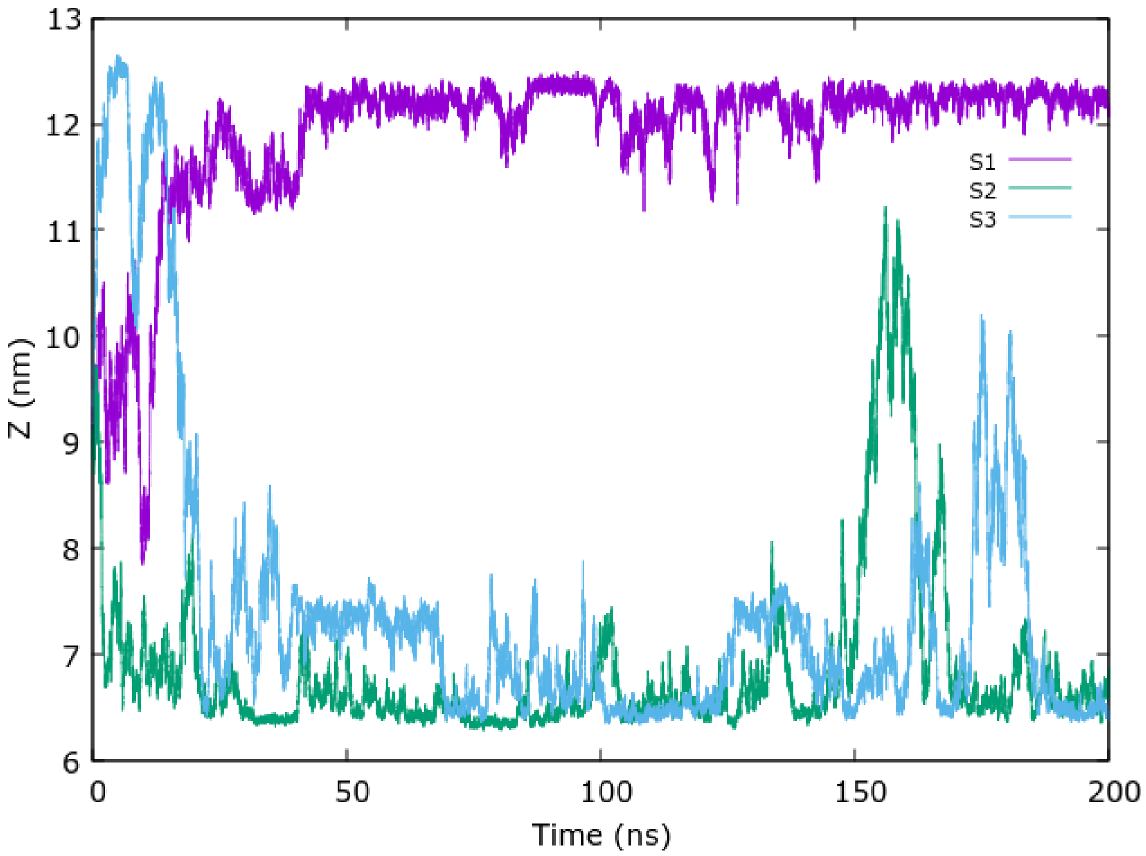

Figure S1: z coordinate of COM of chitosan 6-mer in triplicate

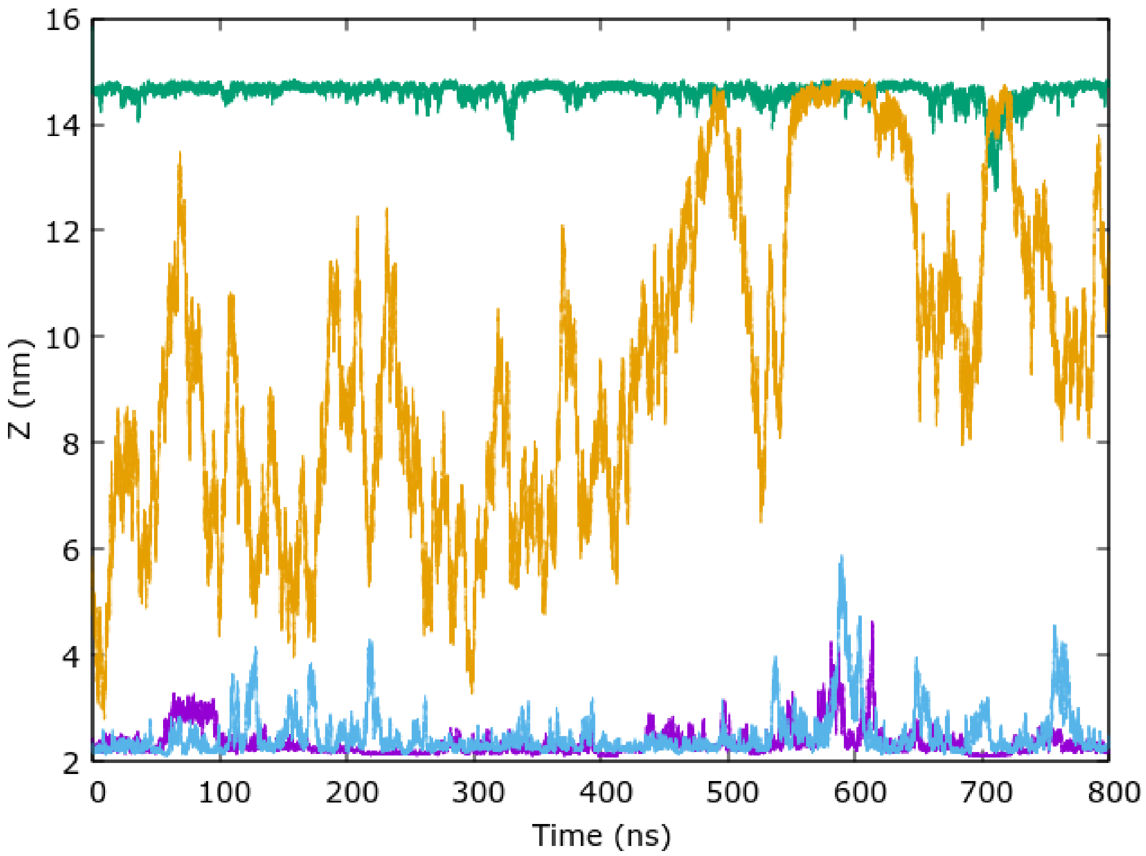

Figure S2: z coordinate of COM of chitosan 10-mer. System wih 4 chitosan chains in solution, which were added one-by-one.

SMD and Umbrella sampling

Table S2: SMD and US system summary

Chitosan	N (water)	N(Na^+)	N(Cl^-)
6-mer	29583	119	89
10-mer	30333	109	83

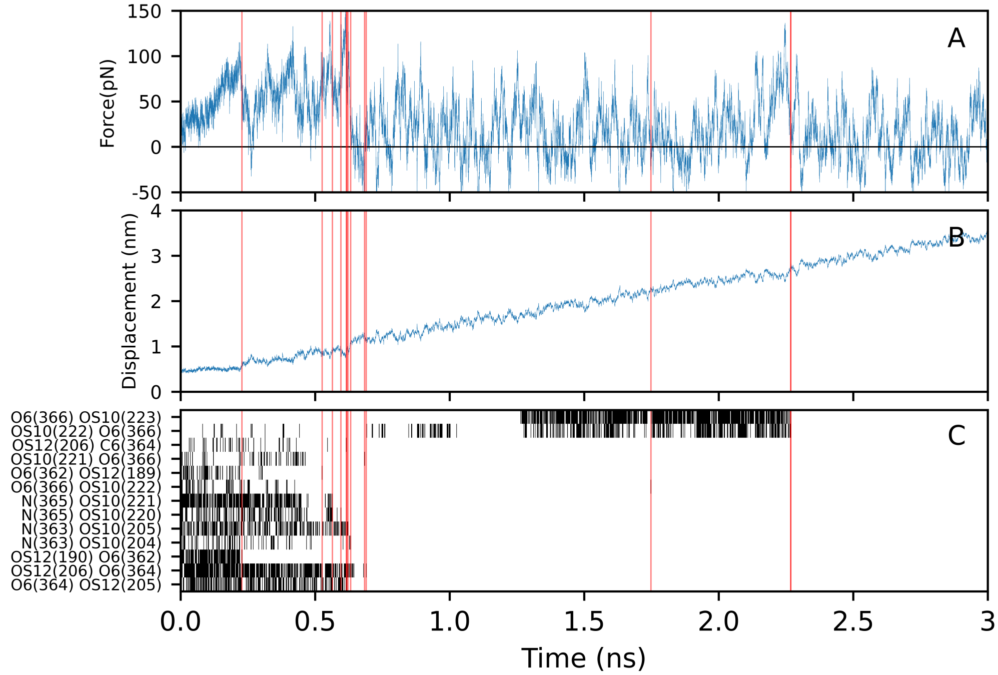

Figure S3: SMD analysis for a 6-mer chitosan oligomer pulled from the silica surface showing (A) the force and (B) the displacement curves. (C) shows when key hydrogen bonds exist, with red vertical lines indicating the time of bond breaking. The pairs are listed in donor-acceptor order. The atom names are as illustrated in Figure 1 in the main text, with the number in the bracket referring to the residue number

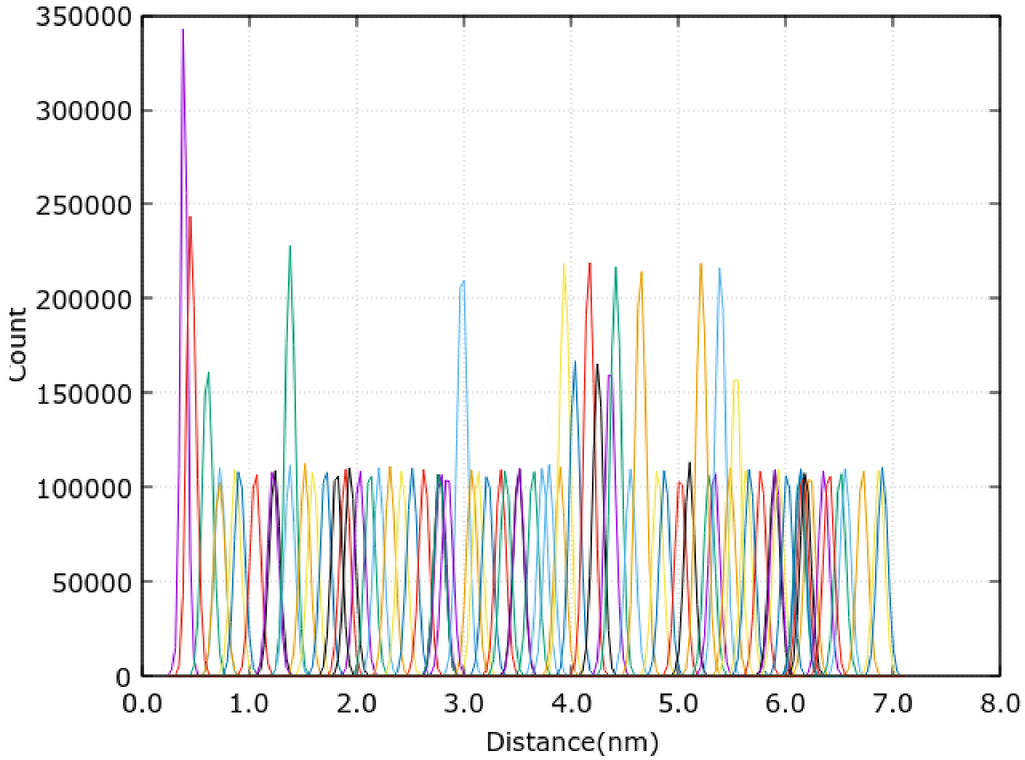

Figure S4: Histograms for 10-mer umbrella sampling set

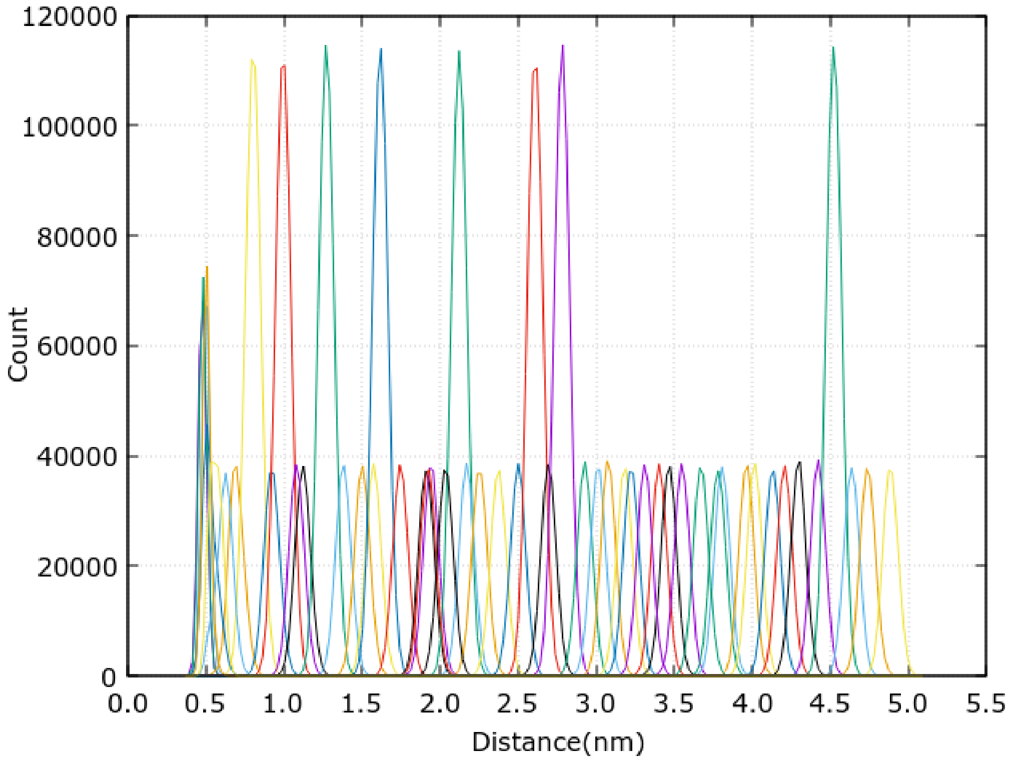

Figure S5: Histograms for 6-mer umbrella sampling set
